# Influence of Carbon Sources on Biomass and Biomolecule Accumulation in *Picochlorum* sp. Cultured under the Mixotrophic Condition

**DOI:** 10.3390/ijerph19063674

**Published:** 2022-03-19

**Authors:** Rahul Kumar Goswami, Sanjeet Mehariya, Obulisamy Parthiba Karthikeyan, Pradeep Verma

**Affiliations:** 1 Bioprocess and Bioenergy Laboratory, Department of Microbiology, Central University of Rajasthan, Kishangarh 305817, India; 2019phdmb10@curaj.ac.in; 2 Department of Engineering, University of Campania “Luigi Vanvitelli”, Real Casa dell’Annunziata, Via Roma 29, 81031 Aversa, Italy; sanjeet.mehariya@unicampania.it; 3 Department of Engineering Technology, College of Technology, University of Houston, Houston, TX 77400, USA; parthibakarthikeyan.obulisamy@sdsmt.edu; 4 Institute of Bioresource and Agriculture, Hong Kong Baptist University, Hong Kong, China; 5 Department of Civil and Environmental Engineering, South Dakota School of Mines and Technology, Rapid City, SD 57701, USA

**Keywords:** *Picochlorum* sp., biomass, lipids, total carotenoids, astaxanthin, β-carotene, sodium acetate

## Abstract

The major downfalls of the microalgal biorefinery are low volume of high value product accumulation, low biomass productivity and high cultivation costs. Here, we aimed to improve the biomass productivity of the industrially relevant *Picochlorum* sp. BDUG 100241 strain. The growth of *Picochlorum* sp. BDUG 100241 was investigated under different cultivations conditions, including photoautotrophic (with light), mixotrophic (1% glucose, with light) and heterotrophic (1% glucose, without light). Among them, *Picochlorum* sp. BDUG100241 showed the highest growth in the mixotrophic condition. Under different (1%) carbon sources’ supplementation, including glucose, sodium acetate, glycerol, citric acid and methanol, *Picochlorum* sp. BDUG100241 growth was tested. Among them, sodium acetate was found to be most suitable carbon source for *Picochlorum* sp. BDUG 100241 growth, biomass (1.67 ± 0.18 g/L) and biomolecule productivity. From the different concentrations of sodium acetate (0, 2.5, 5.0, 7.5 and 10 g/L) tested, the maximum biomass production of 2.40 ± 0.20 g/L with the biomass productivity of 95 ± 5.00 mg/L/d was measured from 7.5 g/L in sodium acetate. The highest total lipid (53.50 ± 1.70%) and total carotenoids (0.75 ± 0.01 µg/mL) contents were observed at the concentration of 7.5 g/L and 5.0 g/L of sodium acetate as a carbon source, respectively. In conclusion, the mixotrophic growth condition containing 7.5 g/L of sodium acetate showed the maximum biomass yield and biomolecule accumulation compared to other organic carbon sources.

## 1. Introduction

Amid the pressing global energy crisis and fossil fuels, negative impacts to the environment required alternatives to fossil fuels [1]. To seek alternative sources of bioenergy, several studies have been carried out, and among them, microalgae can be considered as a potential feedstock for the production of bioenergy products [1]. Microalgae are unicellular, eukaryotic and photosynthetic microorganisms with a rich source of lipids and carbohydrates, and they can be used to produce valuable products including bioenergy and biofuels. Moreover, their biomass also contains pigments, such as chlorophyll, proteins, total carotenoids, β-carotene and astaxanthin, which have great applications in functional foods, pharmaceuticals, cosmetics and the feed industry [2]. The applications of microalgae are hampered by the huge production costs associated with the growth rate and biomass production, harvesting time and cellular composition of biomolecules and use of energy-intensive harvesting methods [3,4]. Therefore, studies on microalgae cultivation and harvesting have received great attention in recent years to reduce the cost of production and improve biomass yields [5].

Different studies have been reported regarding the enhancement of the microalgal biomass and biomolecules, such as the optimization of physiological and environmental conditions, nutritional and physical stress, using the organic waste, modification in the cultivation vessels or systems, etc. [6]. Microalgae can be cultivated under photoautotrophic, heterotrophic and mixotrophic modes using different carbon sources. The phototrophic growth of microalgae is a natural growth mode that uses CO_2_ as a carbon source in the presence of light, but it has low biomass [7]. Additionally, Vega et al. [8] cultivated *Picochlorum* sp. in phototrophic mode and observed that biomass production was low (1.8 g/L). Heterotrophic microalgae (dark mode with the presence of organic or inorganic carbon sources) cultivation can increase the growth rate and thereby biomass productivity. For example, Marudhupandi et al. [9] used a heterotrophic method for the cultivation of *Nannochloropsis salina* using different carbon sources. The maximum biomass was achieved 1.85 g/L in the medium containing glucose (7.959 g/L), sodium acetate 1.46 g/L, peptone (7.6 g/L) and sodium thiosulphate (1.05 g/L) as carbon source. However, most microalgae are unable to survive under heterotrophic growth conditions. Furthermore, the mixotrophic cultivation mode (supplementation of organic and inorganic carbon sources in the presence of light conditions) showed a high biomass production compared to the photoautotrophic and heterotrophic cultivation mode. For example, Gupta and Pawar [10] used acetate as carbon sources for the cultivation of *Scenedesmus abundans*, where the highest biomass and lipid productivities were 59 ± 2.0 and 17 ± 1.8 mg/L/day, respectively. Similarly, Huang et al. [5] also used glucose and acetate for the mixotrophic cultivation of *Chlorella sorokiniana*. It is suggested that optimization of medium components are the main approaches to improve biomass and biomolecules’ productivity. In mixotrophic cultivation conditions, carbon sources play an important role in the development of mixotrophic conditions to achieve the highest biomass and lipid production. Inorganic carbon sources, such as carbon dioxide (CO_2_) and HCO_3_^−^, have desirable qualities to contribute to lowering the risk of medium contamination by unwanted microorganisms. However, it is difficult for algal cells to achieve high biomass productivity [1]. Moreover, various organic carbon sources including glucose, glycerol, citric acid, methanol and sodium acetate have been explored to maximize biomass production under mixotrophic cultivation conditions. Among them, sodium acetate is the most used organic carbon due to its lower cost, yet it is the most easy to assimilate as an energy source by microalgae [1]. Specifically, sodium acetate improves the metabolic process, where acetyl-CoA catalyzes the formation of acetyl-CoA from acetate in algal cells and participates in the metabolism of the citric acid cycle for lipid synthesis. Additionally, it improves carbon metabolic flux in the intracellular citric acid cycle, which supports growth and biomass productivity. However, the optimal concentration of sodium acetate varies among different microalgal strains, which requires thorough testing [1].

Moreover, integrated biorefinery approaches such as the production of pigments and high value-added biomolecules facilitate a more economic process compared to single biofuels feedstock production. Therefore, potential microalgal strains need to be explored for the integrated biorefinery, which can produce a significant number of biofuels as well as various high value-added metabolites [2]. Among various algal strains *Picochlorum* sp. is recognized as an efficient strain because of its physiological properties, i.e., broad thermotolerant (stable up to 40 °C), salinity tolerance (0.2–1 M NaCl) [11,12] and high biomass production (~1.50–2.40 g/L^−^) [13] and a faster exponential growth rate (0.05–0.15/h) [11,12] as compared to *Dunaliella* and *Nannochloropsis* [14,15]. On the other hand, biomass contains high lipids (20–58%) and total carotenoids (0.9–8.2 mg/g) [15] that can be sustainable microalgae for integrated biorefinery [16]. Only very limited studies have been explored to examine the potential of *Picochlorum* sp. to produce lipids and high value-added metabolites.

In this study, the potential of *Picochlorum* sp. BDUG 100241 was explored under a mixotrophic cultivation condition to improve biomass yield and biomolecule production. The effect of different organic carbon sources including glucose, acetate, glycerol, methanol and citric acid were accessed in mixotrophic growth conditions to improve the lipids, chlorophyll, total carotenoids and astaxanthin and β-carotene accumulation by *Picochlorum* sp. BDUG 100241. To the best our knowledge, no reports are available on microalgae strain *Picochlorum* sp. BDUG 100241 or on a comparative study between various cultivation modes such as photoautotrophic, mixotrophic and heterotrophic conditions. Additionally, the effects of different carbon sources under mixotrophic conditions were also studied. Therefore, this study will provide a new approach to improve biomass and biomolecule production. Furthermore, it helps to design the large-scale cultivation of microalgae biomass and helps to design the integrated biorefinery for the production of valuable bioproducts and biofuels.

## 2. Materials and Methods

### 2.1. Culture Collection, Culturing and Microscopic Observation

*Picochlorum* sp. BDUG 100241 was purchased from the cultural collection center of Bharathidasan University, Department of Marine Biotechnology, Tiruchirappalli, Tamilnadu, India. *Picochlorum* sp. BDUG 100241 was subcultured in artificial seawater nutrient III (ASN-III) media and incubated for 20 days at 25 °C. After incubation, the seed culture was stored at 4 °C for further use. The microscopic observation (40× and 100×) was performed using a Zeiss Lab A1 microscope, from Germany.

### 2.2. Experimental Methodology

#### 2.2.1. Media Selection and Growth Phase

Three different types of media—ASN-III [17], Blue-green 11 (BG-11) [17], and Modified Johnson (MJ) [18]—were prepared, and pH was adjusted to 7.0 using 0.1 M acid/alkali. *Picochlorum* sp. BDUG100241 was cultured in a 250 mL Erlenmeyer flask containing 50 mL of different types of media. In each culture flask, 10% of inoculum (OD: ~0.1) was added [14]. The culture flask was incubated for 30 days at 25 °C in a stable culture rack with a continuous light intensity of 3000 lux to observe the growth phase. 

#### 2.2.2. Establishment of Mixotrophic Growth Mode

It is necessary to determine whether the *Picochlorum* sp. could grow in mixotrophic and heterotrophic cultivation (growth) conditions. So, different growth conditions for *Picochlorum* sp. BDUG100241 need to be established. For heterotrophic cultivation mode, the 250 mL Erlenmeyer flask contained 50 mL of ASN-III medium with 1% glucose and was incubated in dark conditions. For mixotrophic mode, the ASN-III medium with 1% glucose was incubated in light and dark conditions (12:12). For photoautotrophic mode (control mode), the ASN-III medium without glucose was incubated in continuous illumination [19]. The incubation period was 20 days for each growth mode. The growth conditions such as temperature, pH and light intensity were the same as mentioned in Section 2.2.1.

#### 2.2.3. Effects of Different Carbon Sources in *Picochlorum* sp.

Different organic carbon sources, such as glucose, glycerol, methanol and sodium acetate, were added (at 1% of *v/v*) to the ASN-III medium to study the effects in the growth, biomass, lipids and pigment production in *Picochlorum* sp. BDUG100241 under mixotrophic conditions. The growth conditions are detailed in Section 2.2.1.

#### 2.2.4. Effects of Different Concentrations of Sodium Acetate in *Picochlorum* sp.

After the selection of a suitable carbon source, its concentration needs to be optimized. Thus, different concentrations of 0, 2.5, 5.0, 7.5 and 10.0 g/L of sodium acetate were added in the culture medium to study the effects of organic carbon load on *Picochlorum* sp. BDUG100241. The biomass and biomolecule extraction and quantification were performed as detailed in the below sections.

### 2.3. Analytical Methods

#### *Picochlorum* sp. BDUG 100241 Growth, Biomass Yield and Productivity

The biomass concentration and growth curve were determined by measuring absorbance at 750 nm at 2-day intervals [16] with a visible-spectrophotometer (LABTRONICS LT-39). For biomass production, a 1 mL sample was centrifuged at 3381× *g* for 10 min, dried in pre-weighed microfuge vials and weighed after drying at 60 °C for 24 h [20]. The biomass production and biomass productivity were calculated by Equations (1) and (2) [21,22].

(1) Biomass yield (g/L)=Final microalgal biomass−Initial microalgal biomass

(2) Biomass productivity (mg/L/d)=(DCW (g/L)×1000)⁄(cultivation time (Days))

where DCW is the dry cell weight.

### 2.4. Harvesting, Extractions and Determination of Lipids and Pigments

#### 2.4.1. Harvesting of *Picochlorum* sp. Biomass

*Picochlorum* sp. BDUG100241 biomass was harvested from the culture by centrifugation at 3019× *g* for 10 min. The harvested biomass was frozen at −20 °C for 24 h. The frozen biomass was lyophilized using Lyophilizer (Labconco Benchtop Lyophilizer, Mumbai, India).

#### 2.4.2. Lipid Extraction and Quantification

The lipid extraction procedure was adapted from [23] and modified according to laboratories conditions and calculated gravimetrically. The dried algal (100 mg) biomass was taken and 2 mL of chloroform, 4 mL of methanol and 1 mL of distilled water in the ratio of 1:2:0.5 were added to the flask [24]. The mixture was continuously stirred for 1 h. After 1 h of extraction, 3 mL of chloroform and 3 mL of methanol were added to the mixture. The mixture was centrifuged at 4602× *g* for 10 min, and after centrifugation, the organic phase was taken and stored in a new tube. Then, 0.5 mL of KOH (5%) and NaOH 0.5 mL (0.76%) were added to the tube and allowed to have phase separation, and then the lower phase was taken. The extraction procedure was repeated until the remaining biomass color was lost. The solvent was evaporated by using a Rota evaporator, from Heidolph, India. The total lipid content was gravimetrically quantified [24].

#### 2.4.3. UV-Spectrometric Analysis of Pigments

For UV-spectrophotometric analysis of microalgal pigments, 1 mL of *Picochlorum* sp. BDUG 100241 photoautotrophically grown culture was taken in a microcentrifuge tube and centrifuged at 2500× *g* for 5 min. The pellet was washed 2 times by adding 1 mL distilled water and repeating the centrifuge process. After the centrifugation, 1 mL of absolute methanol and 1 mL of hexane were added and incubated for 24 h at room temperature. After incubation, the tube was vortexed for 15 s and then centrifuged at 1500× *g* for 5 min [25,26,27]. The supernatant was used for pigment characterization of chlorophyll and β-carotene. For UV-spectrometric analysis of astaxanthin, we similarly followed the above-mentioned procedure until a pellet was formed. Then, 1 mL of DMSO was added to the pellet and vortex well. After the vortex, the sample was centrifuged at 4602× *g* for 10 min, and the supernatant was used for the characterization of astaxanthin [28]. The UV characterization of *Picochlorum* sp. BDUG 100241 was characterized using Shimadzu UV-2600, Japan, and compared with standard pigments of astaxanthin and β-carotene. 

#### 2.4.4. Extraction of Chlorophyll a and b

The microalgal culture (1 mL) was withdrawn after the end of the log phase. Cells were centrifuged at 1500× *g* for 5 min. The supernatant was discarded. The pellet was washed 2 times by adding 1 mL of distilled water and repeating the centrifuge process. Then, 1 mL of absolute methanol was added and incubated for 24 h at room temperature. After incubation, the tube was vortexed for 15 s and then centrifuged at 1500× *g* for 5 min [25]. Absorbance was taken at 652 and 665 nm for chlorophyll a and b, respectively. The chlorophyll concentration was determined by the extinction coefficient in methanol and calculated by using Equations (3) and (4) [21,22,25].

(3) Chla (µg/mg)=16.72×A665.2−16×A652.4

where chla is chlorophyll a, A665.2 is the absorbance at A665.2 nm and A652.4 is the absorbance at 652.4 nm.

(4) Chlb (µg/mg)=34.09×A652.4−15.28×A665.2

where chlb is chlorophyll b, A652.4 is the absorbance at 652.4 nm and A665.2 is the absorbance at A665.2 nm.

#### 2.4.5. Extraction of Total Carotenoids

The microalgal culture (1 mL) was withdrawn after the end of the log phase. Cells were centrifuged at 1500× *g* for 5 min. The supernatant was discarded. The pellet was washed 2 times by adding 1 mL of distilled water and repeating the centrifuge process. Then, 1 mL of absolute methanol was added and incubated for 24 h at room temperature. After incubation, the tube was vortexed for 15 s and then centrifuged at 1500× *g* for 5 min. [25]. Absorbance was taken at 470 nm using UV-Visible spectroscopy (Shimadzu UV-2600, Kyoto, Japan) and calculated by using Equation (5) [25,29].

(5) Total carotenoids (µg/mg)=((1000×A470−1.63×chla−1.63×104.96×chlb))/221

where A470 is the absorbance at 470 nm, chla is chlorophyll a and chlb is chlorophyll b.

#### 2.4.6. Extraction and Quantification of Astaxanthin

The lyophilized (1.0 mg) *Picochlorum* sp. BDUG100241 biomass was weighed. The biomass was saponified using 1 mL of 30% methanol containing 5% KOH (*w/v*). The sample was vortexed for 2 min and incubated at 70 °C for 5 min to remove the chlorophyll content to avoid any interference. The sample was centrifuged at 3020× *g* for 10 min. The obtained pellet was used for the extraction of astaxanthin using 1 mL DMSO. The sample was strongly vortexed and incubated at 70 °C for 10–40 min. After the incubation period, the sample was centrifuged at 4602× *g* for 10 min. The supernatant was used for the quantification of astaxanthin using a UV-visible spectrophotometer at 490 nm. The following Equation (7) was used for the estimation of total astaxanthin [28].

(6) Astaxanthin (µg/mg)=((4.5×A490×volume of extract))/(Weight of sample)

where A490 is the absorbance at 490 nm.

#### 2.4.7. Extraction and Quantification of β-Carotene

The lyophilized (1.0 mg) *Picochlorum* sp. BDUG100241 biomass was weighed and 2 mL of ethanol, 1 mL of hexane and 2 mL of water were added. The sample was vortexed well and centrifuged at 1087× *g* for 10 min. After the centrifugation, the supernatant phase was divided into two phases. The upper phase (hexane phase) was used for the determination of β-carotene using a UV-visible spectrophotometer at 450 nm. The amount of β-carotene was calculated using Equation (7) [23]

(7) β−carotene (µg/mg)=(25.2×A450)

where A450 is the absorbance at 450 nm.

### 2.5. Statistical Analysis

All experiments and analysis in the study were performed in biological replicates and technical triplicate. The results are expressed as the mean ± standard deviation.

## 3. Results and Discussions

### 3.1. Microscopic Observation and Morphology

**Figure 1:**
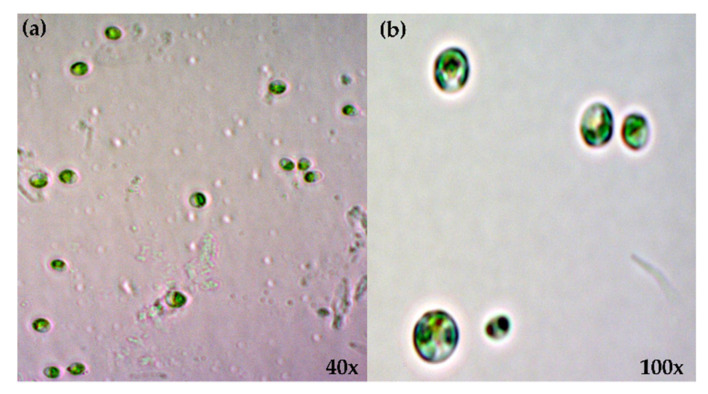
Representative microscopic image of *Picochlorum* sp. BDUG100241 (**a**) 40× and (**b**) 100× magnification under the photoautotrophic condition.

The 40× and 100× image analysis of *Picochlorum* sp. BDUG100241 shown in Figure 1 suggests that it is a small, green, unicellular, coccoid-shaped microalgal containing chlorophyll in the cells. *Picochlorum* sp. BDUG 100241 is morphologically the same as that of the other reported *Picochlorum* sp. The cells generally represent a single chloroplast with no pyrenoids [15].

### 3.2. Determination of Pigment Present in Picochlorum sp. Biomass

**Figure 2:**
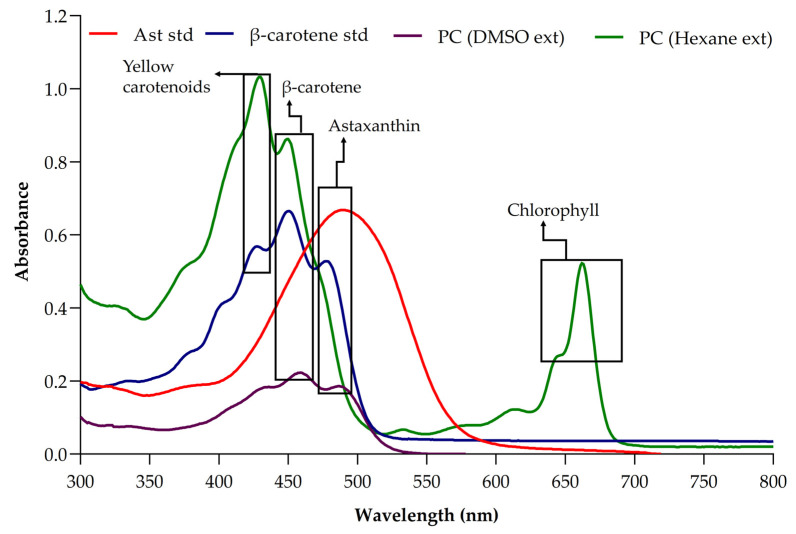
UV characterization of different pigments present inside the *Picochlorum* sp. BDUG 100241 biomass extracts under the photoautotrophic condition; astaxanthin standard (Ast std), β-carotene standard (β-carotene std), *Picochlorum* sp. DMSO extract (PC (DMSO ext)) and *Picochlorum* sp. hexane extract (PC (Hexane ext)).

In photoautotrophic conditions, *Picochlorum* sp. BDUG100241 biomass contains different pigments, such as chlorophyll, β-carotene and yellow carotenoids (lutein), in a combination of hexane and methanol extract at the wavelength of 652–665 nm, 450 nm and 440 nm, respectively (Figure 2). Vega et al. [8] used methanol for the preparation of *Picochlorum* sp. HM1 biomass extract, which showed the presence of yellow carotenoids (lutein in their biomass). Furthermore, Cano et al. [30] also reported that the *Picochlorum celeri* contains β-carotene, chlorophyll and canthaxanthin in an 80% methanol and 20% acetone extract mix. Similarly, Barten et al. [31] also reported the presence of carotenoids and chlorophyll in *Picochlorum* sp. They studied the short-term physiological responses in *Picochlorum* sp. using different optimal temperatures for the accumulations of pigments. However, the present study suggested the presence of high concentrations of yellow carotenoids and β-carotene accumulation. Furthermore, DMSO extract of *Picochlorum* sp. BDUG100241 showed the presence of astaxanthin at the wavelength of 490 nm (Figure 2). Only very limited studies have reported on the presence of astaxanthin from the *Picochlorum* sp. biomass [30,31,32]. These reported findings strongly supported the results obtained in this study, which contained different carotenoids in *Picochlorum* sp. BDUG100241.

### 3.3. Impact of Cultivations Media and Growth Conditions in Picochlorum sp. BDUG100241

**Figure 3:**
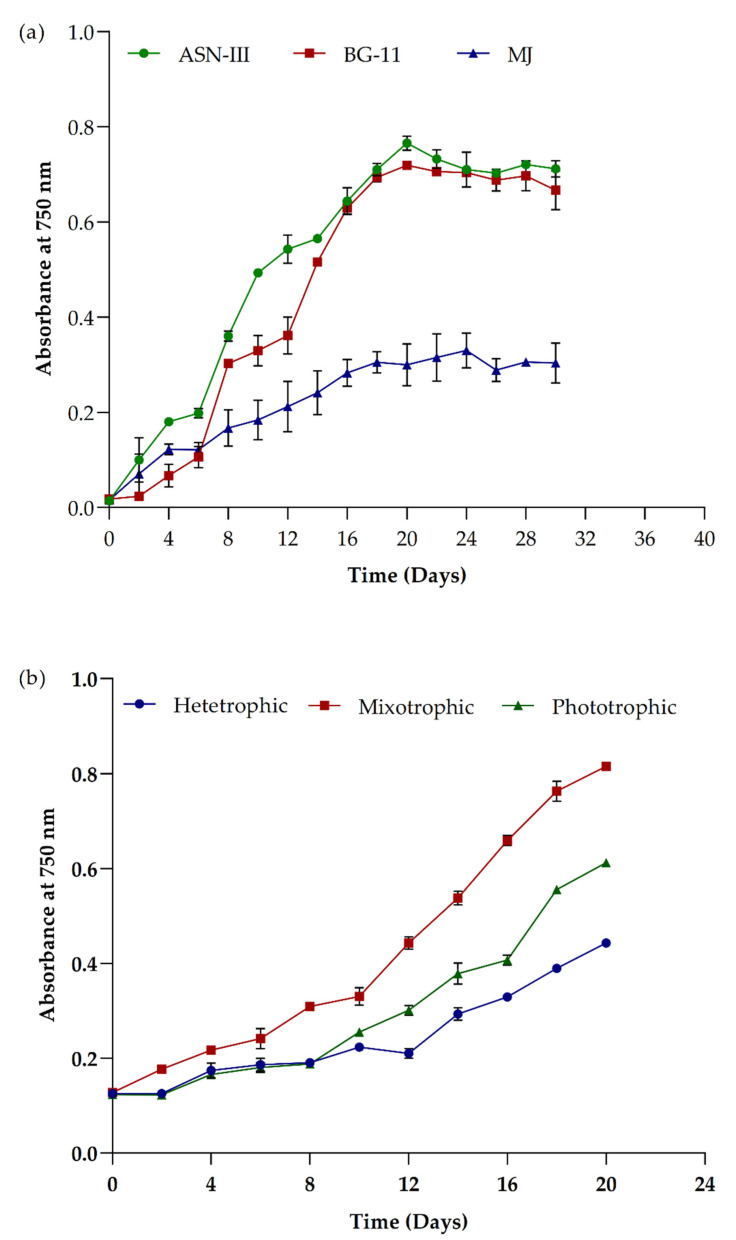
Impact of (**a**) different cultivation media and (**b**) growth modes in *Picochlorum* sp. BDUG 100241.

Different cultivation media (ASN-III, MJ, BG-11) were used to access the growth pattern of *Picochlorum* sp. BDUG100241 under the photoautotrophic mode. As shown in Figure 3a, in ASN-III and BG-11, the *Picochlorum* sp. BDUG100241 showed a similar growth pattern and highest absorbance at 750 nm after the 20-day cultivation period. On the other hand, the MJ media showed a longer stationary phase, but low absorbance and growth compared to other media. Based on this preliminary assessment and the data obtained, the ASN-III medium was found to be more suitable for *Picochlorum* sp. BDUG100241 cultivation than other conventional media, i.e., BG-11 or MJ. Therefore, ASN-III was used for further testing and optimization of growth conditions for *Picochlorum* sp. BDUG100241. Moreover, no literature has been reported that studied the effects of different cultivation media on the growth of *Picochlorum* sp. BDUG100241.

In a subsequent study, three different cultivation modes, i.e., photoautotrophic (light, ASN-III media) heterotrophic (1% glucose in ASN-III media, without light) and mixotrophic (1% glucose in ASN-III media, with light), were tested. As shown in Figure 3b, the mixotrophic condition (1% of glucose, with light) showed a twofold higher biomass growth than photoautotrophic and heterotrophic conditions. Similar reports were published earlier that showed the mixotrophic cultivation conditions produced the highest biomass [9,14,33,34]. For example, Gupta and Pawar [10] reported that mixotrophic cultivation of microalga *Scenedesmus abudans* showed the highest biomass and pigment production compared to photoautotrophic conditions. Similarly, Huang et al. [5] used sodium acetate and glucose for the development of mixotrophic conditions to cultivate *Chlorella sorokiniana*. The results showed that the addition of acetate and glucose (mixotrophic conditions) improved the lipid and biomass production in microalgae. Similarly, Pang et al. [33] reported that mixotrophic mode promoted the growth and biomass production in microalga *Picochlorum* sp. Furthermore, they also suggested that some of *Picochlorum* sp. cannot grow under complete darkness (heterotrophic conditions) [33]. Therefore, mixotrophic modes of cultivation are ideal for high-rate biomass production; however, it is evident that the effect of different carbon sources on yield and bioproduct accumulation requires further investigation.

### 3.4. Impact of Organic Carbon Sources on Growth and Biomass Yield

**Figure 4:**
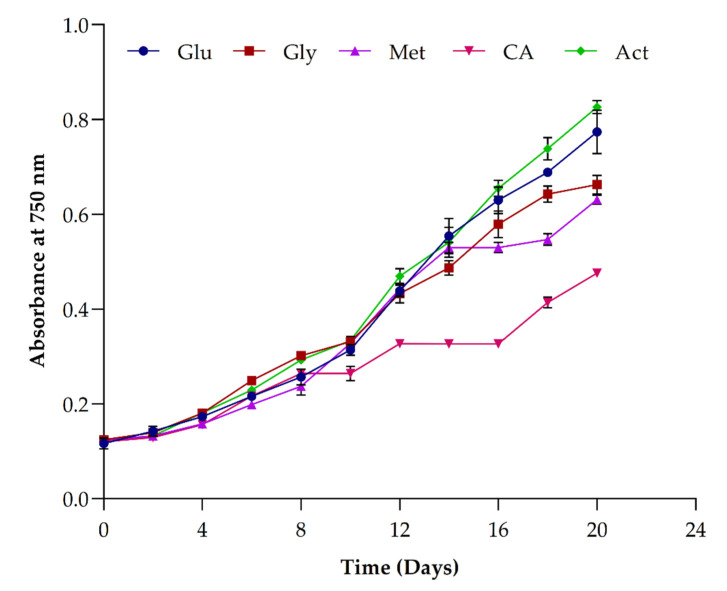
Influence of different organic carbon sources in the growth of *Picochlorum* sp. BDUG-100241; glucose (Glu), glycerol (Gly), methanol (Met), citric acid (CA) and sodium acetate (Act).

The key objective of our study was to access the effects of different organic carbons on *Picochlorum* sp. BDUG100241. Thus, we tested the effect of different organic carbons sources, such as glucose, glycerol, citric acid, methanol and sodium acetate, on *Picochlorum* sp. BDUG 100241 biomass yield under mixotrophic growth conditions. The different carbon sources were tested under mixotrophic conditions, and among them, sodium acetate supports a higher growth rate, as shown in Figure 4. The effects of different carbon sources were also studied by Bhatnagar et al. [19], who reported that supplementation of glucose promoted the growth of microalgae. Similarly, Abreu et al. [35] studied the effects of organic carbon sources for the establishment of a mixotrophic mode and compared the lipid or starch content in the produced biomass of *Chlorella vulgaris*. Furthermore, Lin et al. [36] tested the effects of glucose, fructose, sucrose and xylose for the mixotrophic cultivation of *Chlorella* species. Among them, sucrose showed the highest dried cell weight (~0.45 g/L) and lipid content (~25%) compared to photoautotrophic and heterotrophic conditions. However, none reported the effects of carbon sources, especially sodium acetate on the *Picochlorum* sp. BDUG 100241 biomass yield and biomolecule production. At the end of the cultivation period (20 days), the *Picochlorum* biomass was harvested from the culture medium. As shown in the Table 1, the highest biomass production (1.67 ± 0.18 g/L) and biomass productivity (68.3 3 ± 8.82 mg/L/d) were observed in sodium acetate, followed by glucose (1.30 ± 0.12 g/L and 50.00 ± 6.01 mg/L/d) and glycerol (0.76 ± 0.20 g/L and 22.78 ± 9.77 mg/L/d). While the lowest biomass production and biomass productivity were observed in citric acid (0.68 ± 0.25 g/L and 18.89 mg/L/d) and methanol (0.74 ± 0.17 g/L and 22.22 ± 8.55 mg/L/d). Sodium acetate is the most used organic carbon for the mixotrophic cultivation of microalgae due to its lower cost [1]. Sodium acetate was assimilated by two pathways: (i) the glyoxylate cycle to form malate in glyoxysomes; and (ii) the tricarboxylic acid cycle to form citrate in the mitochondria, which provide the carbon skeletons, energy as ATP and NADH reduction. By these mechanisms, acetate can improve the carbon metabolic flux in the intracellular citric acid cycle and contribute to the increase the biomass production and biomolecule production [10], and a suggested pathway was also deliberated by [1]. Thus, we believe that the *Picochlorum* sp. BDUG 100241 followed one of the pathways and converted organic carbon (sodium acetate) into biomass. Moreover, the lower biomass produced in citric acid might be due to the low bicarbonate alkalinity and pH of the medium. The methanol assimilation metabolic pathway was not well explored for *Picochlorum* sp. BDUG 100241, but it could be noted that the growth was not favored. A similar result was obtained by Bhatnagar et al. [19] where the methanol showed the lowest chla and growth rate in microalgae. Additionally, glycerol was not found to be an ideal carbon source for *Picochlorum* sp. BDUG 100241. However, few reports suggested that supplementation of glycerol improves the biomass production in microalgae. For example, Choi and Yu [37] used crude glycerol for the mixotrophic cultivation of microalgae. The obtained results show that 5.0 g/L of crude glycerol produced more than 1.5 g/L of biomass in microalgae. However, Skorupsikaite et al. [38] reported that the addition of glycerol (2.70 g/L) influenced the biomass production (2.41 g/L) in *Chlorella* sp. Our obtained result showed that, in *Picochlorum* sp. BDUG100241, glycerol does not have a higher impact on biomass productivity as compared to sodium acetate. Overall, sodium acetate or glucose was found to be a better carbon source for the cultivation of *Picochlorum* sp. BDUG 100241 under mixotrophic growth conditions.

### 3.5. Impact of Organic Carbon Sources on Lipids and Pigments’ Accumulation

The lipids are important biomolecules accumulated by microalgae cells, which are used as feedstock for biodiesel production [39]. Moreover, mixotrophic cultivation also influences the accumulation of lipids inside the microalgal cells [36]. For example, Lin et al. [36] developed different cultivation conditions to test the lipid production in microalgae *Chlorella* sp. Y8-1. Their result showed that mixotrophic cultivation promoted the accumulation of lipids in *Chlorella* sp. Y8-1 compared to the autotrophic and heterotrophic conditions. Similarly, Lin et al. [36] compared lipid production in different cultivation modes. The results showed that the addition of 4 g/L of glucose in mixotrophic conditions promoted the 1.8–2.4-fold and 5.2–5.4-fold higher lipid content compared to heterotrophic and photoautotrophic conditions respectively. In the present study (Table 1), the *Picochlorum* sp. BDUG 100241 produced the highest lipid content per mg dry cell weight (DCW) in the supplementation of 1% glucose, i.e., 52 ± 2.8% followed by sodium acetate (51 ± 2.8%) and glycerol (51 ± 1.4%). Additionally, methanol- and citric acid-supplemented biomass also contains 50 ± 2.8% of lipid content. These obtained results showed that the lipid accumulation potential of *Picochlorum* sp. BDUG 100241 was quite varied among all organic carbon sources. Moreover, nutrient or physiological stress conditions can promote the accumulation of lipids inside the microalgal cells. However, the optimized carbon concentrations with light stress can be a possible route to improve the lipid contents in *Picochlorum* sp. biomass.

Similarly to biomass production, chlorophylls are also an important parameter to measure the growth of microalgae. The cultivation conditions also have an impact on the biosynthesis of chlorophyll. For example, Kong et al. [40] compared the mixotrophic condition with other cultivations conditions. Their obtained results showed that mixotrophic cultivation modes promoted a twofold higher biosynthesis of chlorophyll compared to photoautotrophic and heterotrophic conditions. Similarly, our studied results showed the effects of different carbons sources (under mixotrophic conditions) for the biosynthesis of chlorophyll. The obtained results showed that chla (2.68 ± 0.29 µg/mL) and chlb (0.40 ± 0.046 µg/mL) were present in the highest amount in sodium acetate followed by glucose. While the lowest chla and chlb were produced in citric acid and methanol. In contrast, Bhatnagar et al. [19] reported that the supplementation of organic acids leads to the maximum chl production (2.32–9.56 mg/L) in microalgae. Similarly, Wen et al. [41] reported that supplementation of sodium acetate enhanced the chlorophyll content in *Haematococcus* sp. However, Cano et al. [30] reported that *Picochlorum celeri* biomass contains <0.5 µg/mL of total chlorophyll. While the reported data showed *Picochlorum* sp. BDUG 100241 biomass contains higher chlorophyll content compared to *Picochlorum celeri* or other *Picochlorum* sp.

The highest total carotenoids were observed in glucose (0.71 ± 0.10 µg/mL) followed by sodium acetate (0.71 ± 0.06 µg/mL) and methanol (0.60 ± 0.06 µg/mL). While the lowest total carotenoids were observed in citric acid (0.60 ± 0.06 µg/mL). Furthermore, astaxanthin (0.64 ± 0.04 µg/mg dry cell biomass weight) and β-carotene (2.72 ± 0.04 µg/mg dry cell biomass weight) were produced in 1% methanol and citric acid supplementation. Vega et al. [8] reported that the *Picochlorum* sp. HM1 biomass contained ~1 mg/g DW of β-carotene, which is slightly lower than the compared result obtained in this study (2.72 ± 0.04 µg/mg). The study suggested that sodium acetate supplementation improved the production of the highest biomass, chlorophyll and total carotenoids production [42]. Additionally, optimization of carbon concentration can improve the lipid content in *Picochlorum* sp. BDUG 100241.

### 3.6. Impact of Different Concentrations of Sodium Acetate

#### 3.6.1. Impact on Biomass Production

**Figure 5:**
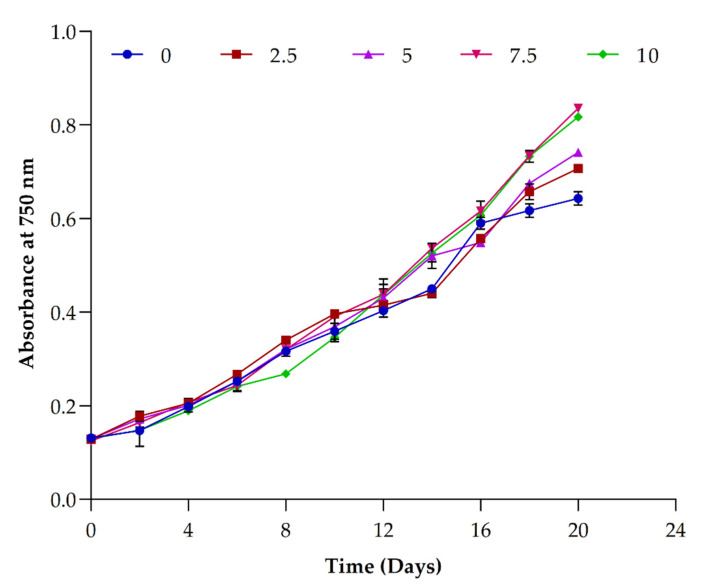
Influence of different concentrations of sodium acetate (0–10 g/L) in *Picochlorum* sp. BDUG 100241 growth under mixotrophic condition.

**Figure 6:**
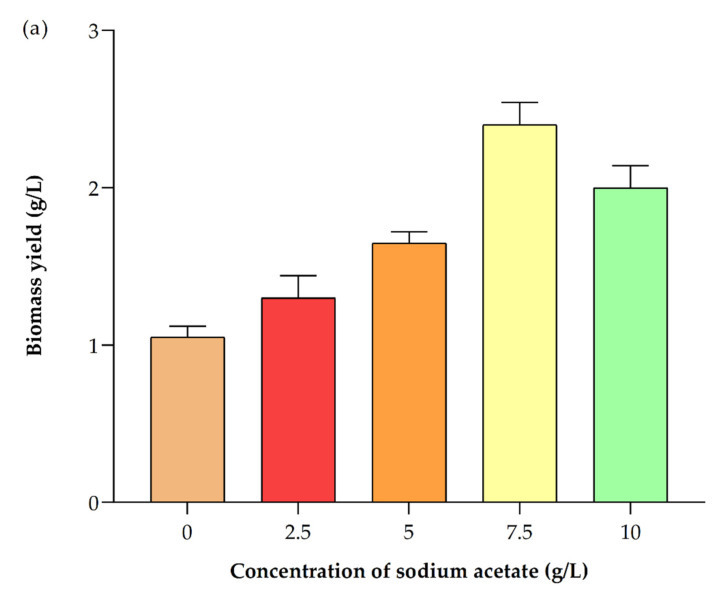

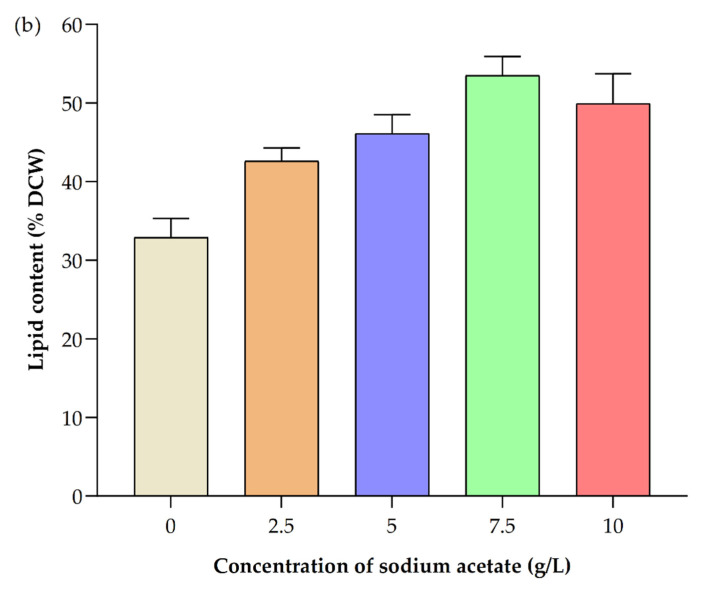
Impact of different concentrations of sodium acetate on *Picochlorum* sp. BDUG 100241 under mixotrophic conditions: (**a**) biomass production and (**b**) lipid content.

It is suggested that the optimal concentration of sodium acetate for the algal growth is distinct among different strains [1]. For example, Rai et al. [42] used different concentrations of sodium acetate and glycerol for the cultivation of *Chlorella pyrenoidosa*. Their results showed that a concentration of 10 g/m^2^ of sodium acetate promoted the sixfold higher biomass in *Chlorella pyrenoidosa*. Thus, we also studied the different concentrations of sodium acetate (0, 2.5, 5.0, 7.5 and 10 g/L) supplemented in an ASN-III medium to study the effects on biomass and biomolecule production in *Picochlorum* sp. BDUG 100241 under mixotrophic conditions. Figure 5 shows that the highest growth was observed at the concentration of 7.5 g/L of sodium acetate. After the end of the cultivation period, a high biomass yield (2.40 ± 0.14 g/L) and production rate (95.00 ± 7.07 mg/L/d) was achieved with the addition of 7.5 g/L of sodium acetate, which was ~1.5- and 2-fold higher than 2.5 and 0 g/L sodium acetate, respectively (summarized in Table 2 and seen in Figure 6a). The carbon mass balance of *Picochlorum* sp. biomass yield was ~0.32 g/g NaAcetate. Garcia et al. [43] used different organic carbon sources including sodium acetate for the cultivation of microalgae. The obtained results showed that the addition of 0.5 M of sodium acetate showed the highest biomass production of 1.15 g/L. Marudhupandi et al. [9] supplemented 1.46 g/L of sodium acetate in the medium for the cultivation of *Nannochloropsis salina*. At the end of cultivation, 1.85 g/L biomass was obtained. Additionally, Cheng et al. [1] also used 0–250 mg/L of sodium acetate for the cultivation of microalgae *Scenedesmus obliquus*. The result showed a biomass yield of 648.057–769.566 mg/L and a biomass production rate of ~31.708–46.997 mg/L/d. Huang et al. [5] used the combination of glucose (5 g/L) and acetic acid (1 mL). The reports suggested that the addition of glucose and sodium acetate improves biomass production ~0.6–2 g/L. Similarly, Gupta and Pawar [10] supplemented 0.5 to 4 g/L of sodium acetate to enhance biomass production. At the concentration of 3 g/L sodium acetate, microalgae showed the highest production of biomass, at ~340 mg/L, compared to other concentrations. However, our study results showed a higher biomass yield and biomass production rate (2.40 ± 0.14 g/L and 95.00 ± 7.07 mg/L/d) with the addition of sodium acetate as a carbon source compared to other published reports for microalgae. However, additions of higher concentrations of carbon sources also decreased biomass production by following feedback inhibition [44]. That is the reason why the *Picochlorum* sp. BDUG100241 showed a lower biomass in 10 g/L of sodium acetate compared to 7.5 g/L of sodium acetate.

#### 3.6.2. Impact on Lipid Production

The addition of sodium acetate as a carbon source under mixotrophic conditions improved the lipid accumulation rate in *Picochlorum* sp. BDUG 100241. Similar results have been published earlier, which suggest that the optimization of sodium acetate can promote the lipid content in microalgae [45,46]. For example, the combination of glucose with sodium acetate promoted the lipid content in *Chlorella* sp. (60% DCW) [5]. Similarly, Gupta and Pawar [10] studied the effect of different concentrations of sodium acetate on lipid accumulation in *Scenedesmus abundans*. They reported that the *Scenedesmus abundans* showed the highest lipid productivity (7 mg/L/d) with the sodium acetate concentration of 3 g/L. Similarly, Ghosh et al. [45] reported that 3 g/L of sodium acetate showed the highest lipid productivity of 176.80 ± 68.80 µg/mg. Lu et al. [46] reported that the addition of sodium acetate can produce 8.63% of lipid contents in *Spirulina platensis*. Furthermore, Rai et al. [43] reported that the addition of 10 g/m^2^ sodium acetate promoted a 13.5-fold higher lipid compared to photoautotrophic conditions. Similarly, our obtained results showed that (Figure 6) the concentration of total lipids was at maximum with the 7.50 g/L of sodium acetate addition in *Picochlorum* sp. BDUG 100241. It accumulated ~53.50 ± 2.40% DCW of lipid content, followed by the 10 g/L sodium acetate addition (49.90 ± 3.82% DCW). On the other hand, without the supplementation of sodium acetate, *Picochlorum* sp. BDUG 100241 accumulated 32.90 ± 2.40% of lipid content DCW of biomass [44]. Similar results were reported earlier [47]. For example, Silva et al. [48] reported that the addition of 7.5 g/L sodium acetate and glucose promoted the biomass yield (1.75 g/L) and lipid content (34.4 0 ± 0.80%) in *Neochloris oleoabundans*. However, our obtained results suggest that at the same concentration of sodium acetate *Picochlorum* sp. BDUG100241 showed 53.50 ± 2.40%. However, without the addition of a carbon source, generally, *Picochlorum* sp. biomass contains 20–40% of lipid content [49]. Similarly, the existence of 10–20% of ash-free dry weight lipid content in *Picochlorum renovo* biomass was also reported. Moussa et al. [16] supplemented 10 g/L of glucose in the medium for lipid production in the microalga *Picochlorum* species. During their study, 167.00 ± 6.00 g/kg of lipid production was achieved as biomass. Thus, these published studies suggest that the optimization of the carbon concentration increases the lipid content in microalgae, and support our obtained results.

#### 3.6.3. Impact on Pigments Production

**Figure 7:**
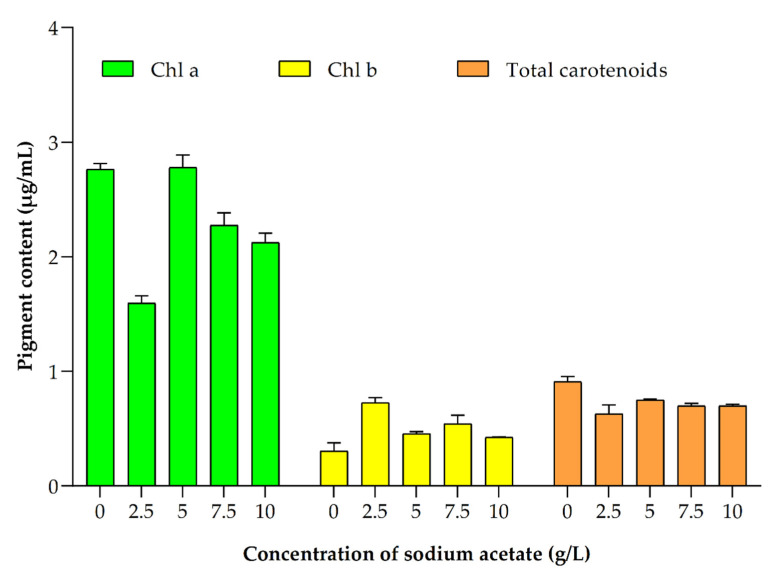
Impact of different concentrations of sodium acetate on *Picochlorum* sp. BDUG 100241 biomass to the production of chla, chlb and total carotenoids under mixotrophic conditions.

In present study, the highest chla was obtained (2.82 ± 0.15 µg/mL) in 7.5 g/L of sodium acetate followed by 5 g/L (2.78 ± 0.15 µg/mL) (Figure 7). Additionally, it was reported that the supplementation of glucose 5 g/L promoted chlorophyll production (~25 µg/mL) compared to 1 g/L of sodium acetate (20 µg/mL) [46]. Moreover, Moussa et al. [16] reported the presence of a high amount of chla (0.50–5.70 mg/g) without the addition of carbons. The results showed that the addition of sodium acetate (0–10 g/L) can promote chla (1.60–2.82 µg/mL) and chlb (0.42–0.73 µg/mL) content. It is reported that the chlb biosynthesis did not show as much variation in all cultivation modes [42] and similar results were obtained by our study.

**Figure 8:**
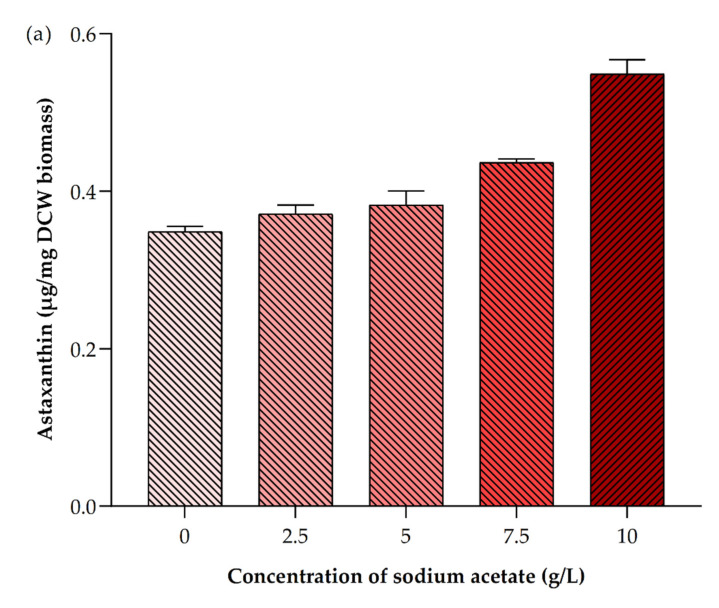

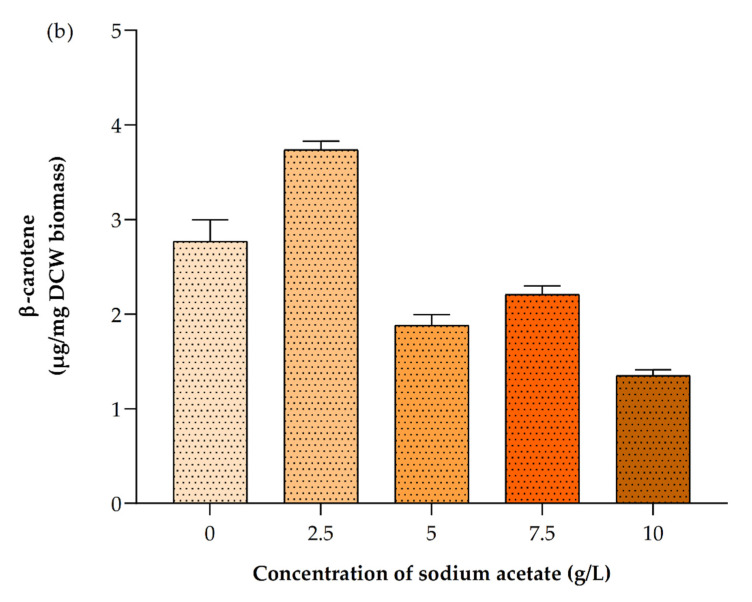
Impact of different concentrations of sodium acetate on *Picochlorum* sp. BDUG 100241 mixotrophic conditions for the accumulation of (**a**) astaxanthin and (**b**) β-carotene.

Carotenoids, such as astaxanthin and β-carotene, were mainly synthesized in the chloroplast, and they have a protective role, working as accessory photosynthetic pigments [27]. Moreover, they have a wide role in the food and pharmaceutical industry due to their health benefits [2,50,51]. Previously, it was reported that the *Picochlorum* sp. biomass contained total carotenoids (0.9–8.2 mg/g). Moreover, different nutrient stress conditions and carbon optimization can increase the total carotenoids content in microalgae [15,52]. Similarly, the present study showed that the supplementation of sodium acetate can also alter the accumulation of total carotenoids in *Picochlorum* sp. BDUG 100241 biomass (0.63 ± 0.11–0.75 ± 0.01 µg/mL) (Figure 7). However, the highest total carotenoids content was observed without the supplementation of sodium acetate (0.91 ± 0.06 µg/mL). Similar results were published earlier. For example, Abreu et al. [35] reported that the photoautotrophic condition showed the highest carotenoids content (0.23%) compared to mixotrophic conditions (0.04–0.08%). Similarly, Wen et al. [42] reported that supplementation of sodium acetate in mixotrophic conditions promoted the total biosynthesis in microalga *Haematococcus* sp. Furthermore, our study showed that supplementation of 2.5 g/L of sodium acetate improved the β-carotene production by 3.74 ± 0.12 µg/mg DCW biomass (Figure 8a). On the other hand, 10 g/L of sodium acetate promoted the astaxanthin accumulation in *Picochlorum* sp. BDUG 100241 (0.55 ± 0.03 µg/mg DCW biomass (Figure 8b). A similar report was found earlier in *Haematococcus* species. For example, Wen et al. [42] cultivated *Haematococcus* sp. under mixotrophic conditions through supplementation of different concentrations of acetate. Their obtained result showed that mixotrophic cultivation promoted 1.2 times higher astaxanthin compared to photoautotrophic conditions.

## 4. Conclusions

*Picochlorum* sp. BDUG100241 is a freshwater green microalga containing lipids and pigments. The biomass of *Picochlorum* sp. BDUG 100241 contains a variety of pigments such as chlorophylls, total carotenoids, β-carotene and astaxanthin, which increases its valuation. This work reported that *Picochlorum* sp. BDUG 100241 showed a higher growth pattern in ASN-III media as compared to other tested media. Compared to photoautotrophic and heterotrophic cultivation conditions, mixotrophic cultivation conditions were found to favor the growth of *Picochlorum* sp. BDUG 100241 and biomolecule accumulations. As we tested the different organic carbon sources, among them wide variation was obtained in their biomass and biomolecule production. Furthermore, the sodium acetate at 7.5 g/L was found to be a more suitable carbon source and citric acid and methanol as the least preferred carbon sources. The optimization of sodium acetate concentration showed variation in their biomass and biomolecule productivity. Moreover, when the concentration of sodium acetate (2.5–7.5 g/L) increased, the biomass (1.30 ± 0.14–2.40 ± 0.14 g/L), lipid content (42.60 ± 1.7–53.50 ± 2.4%), chlorophyll (1.60 ± 0.09–2.82 ± 0.15 µg/mL) and astaxanthin (0.37 ± 0.02–0.55 ± 0.03 µg/mg DCW biomass) also increased. However, when the concentration of sodium acetate increased above the optimized concentration, the biomass and biomolecule production was slightly decreased. Previously reported literature has suggested that this reduction occurs due to feedback inhibition. Thus, this study suggested that, for higher mixotrophic growth, optimum carbons sources are required. Moreover, it can promote the *Picochlorum* sp. BDUG 100241 growth and their optimal concentrations directly influence the biomass and biomolecule composition. Overall, it can be concluded that *Picochlorum* sp. BDUG 100241 is a resilient strain containing lipids as well as pigments. The outcomes of this study suggest that the *Picochlorum* sp. BDUG 100241 strain holds great potential as an integrated platform to produce high-value bioproducts and biofuels. Furthermore, this research has great significance to the sustainable development of large-scale algal biorefinery production using *Picochlorum* sp. BDUG 100241.

## Figures and Tables

**Table 1:** Biomass and biomolecule production in different carbon sources (*n* = 3), ± (standard deviation of replicate data).

Carbon Sources	Biomass Yield (g/L)	Biomass Productivity (mg/L/d)	Lipid Content (%) in Dry Cell Weight (DCW)	Chla (µg/mL)	Chlb (µg/mL)	Total Carotenoids (µg/mL)	Astaxanthin (µg/mg DCW Biomass)	β-Carotene (µg/mg DCW Biomass)
Glu	1.30 ± 0.12	50.00 ± 6.01	52 ± 2.8	2.49 ± 0.21	0.35 ± 0.09	0.71 ± 0.10	0.53 ± 0.01	1.30 ± 0.37
Gly	0.76 ± 0.20	22.78 ± 9.77	51 ± 1.4	1.48 ± 0.03	0.32 ± 0.015	0.56 ± 0.07	0.35 ± 0.02	1.89 ± 0.21
Met	0.74 ± 0.17	22.22 ± 8.55	50 ± 2.8	1.44 ± 0.35	0.30 ± 0.007	0.60 ± 0.06	0.64 ± 0.04	2.46 ± 0.12
CA	0.68 ± 0.25	18.89 ± 12.62	50 ± 2.8	0.92 ± 0.05	0.24 ± 0.013	0.44 ± 0.04	0.37 ± 0.04	2.72 ± 0.04
Act	1.67 ± 0.18	68.33 ± 8.82	51 ± 2.8	2.68 ± 0.29	0.40 ± 0.046	0.71 ± 0.06	0.59 ± 0.02	1.89 ± 0.07

Note: number of replicate (n), glucose (glu), glycerol (gly), methanol (met), citric acid (CA), sodium acetate (Act) and dry cell weight (DCW).

**Table 2:** Impact of different sodium acetate concentrations on *Picochlorum* sp. BDUG 100241 biomass and biomolecule production (*n* = 3, ±standard deviation of replicates).

Sodium Acetate Concentration g/L	Biomass Yield (g/L)	Biomass Productivity (mg/L/d)	Lipid Content (%) in Dry Cell Weight (DCW)	Chla (µg/mL)	Chlb (µg/mL)	Total Carotenoids (µg/mL)	Astaxanthin (µg/mg DCW Biomass)	β-Carotene (µg/mg DCW Biomass)
0	1.10 ± 0.07	27.50 ± 3.54	32.90 ± 2.40	2.76 ± 0.07	0.30 ± 0.10	0.91 ± 0.06	0.35 ± 0.01	2.77 ± 0.32
2.5	1.30 ± 0.14	40.00 ± 7.07	42.60 ± 1.70	1.60 ± 0.09	0.73 ± 0.06	0.63 ± 0.11	0.37 ± 0.02	3.74 ± 0.12
5	1.65 ± 0.07	57.50 ± 3.54	46.10 ± 2.40	2.78 ± 0.15	0.46 ± 0.02	0.75 ± 0.01	0.38 ± 0.03	1.88 ± 0.16
7.5	2.40 ± 0.14	95.00 ± 7.07	53.50 ± 2.4	2.82 ± 0.15	0.54 ± 0.10	0.70 ± 0.03	0.044 ± 0.01	2.21 ± 0.12
10	2.00 ± 0.14	75.00 ± 7.07	49.90 ± 3.82	2.13 ± 0.11	0.42 ± 0.00	0.70 ± 0.02	0.55 ± 0.03	1.35 ± 0.09

## Data Availability

Not applicable.
